# Calixarenes as High Temperature Matrices for Thermally Activated Delayed Fluorescence: C_70_ in Dihomooxacalix[4]arene

**DOI:** 10.3390/molecules23030558

**Published:** 2018-03-02

**Authors:** Tiago Palmeira, Alexandre S. Miranda, Paula M. Marcos, Mário N. Berberan-Santos

**Affiliations:** 1 CQFM-IN and IBB—Institute of Bioengineering and Biosciences, Instituto Superior Técnico, Universidade de Lisboa, 1049-001 Lisboa, Portugal; tiago.palmeira@tecnico.ulisboa.pt (T.P.); miranda.m.alexandre@gmail.com (A.S.M.); 2 Centro de Química Estrutural, Faculdade de Ciências da Universidade de Lisboa, Edifício C8, 1749-016 Lisboa, Portugal; pmmarcos@fc.ul.pt; 3 Faculdade de Farmácia da Universidade de Lisboa, Av. Prof. Gama Pinto, 1649-003 Lisboa, Portugal

**Keywords:** thermally activated delayed fluorescence (TADF), organic light-emitting diodes (OLEDs), fullerene C_70_, homooxacalixarenes

## Abstract

Thermally activated delayed fluorescence (TADF) of ^12^C_70_ and ^13^C_70_ was observed up to 140 °C in a *p-tert*-butyldihomooxacalix[4]arene solid matrix, a temperature range significantly higher than that of previous TADF quantitative studies. An effective singlet–triplet energy gap of 29 kJ/mol and triplet formation quantum yields of 0.97 and 0.99 were measured for ^12^C_70_ and ^13^C_70_, respectively. The photophysical properties of the two fullerenes in this new matrix are comparable to those obtained in polystyrene at a lower temperature range. Calixarenes are proposed to be suitable matrices for high temperature TADF studies and applications.

## 1. Introduction

Thermally activated delayed fluorescence (TADF) is a type of fluorescence emission displaying both singlet and triplet properties: the emission spectrum is like that of normal (prompt) fluorescence, whereas the lifetime is close to that of phosphorescence [1,2]. In the TADF mechanism, after excitation and once the S_1_ state is reached, intersystem crossing (ISC) to the triplet manifold takes place, followed by a second ISC back to S_1_, from which fluorescence ensues. The S_1_-T_1_-S_1_ cycle can occur several times. However, this mechanism is only operative for very low concentrations of molecular oxygen, otherwise the triplet state is quenched [1,2,3,4,5]. Not too low temperatures are also required, as the reverse ISC is thermally activated. For this reason, matrices capable of withstanding high temperatures and simultaneously not quenching TADF are of great interest.

TADF is not observed in most fluorophores—e.g., aromatic hydrocarbons and derivatives—owing to the large singlet–triplet energy gap (ΔE_ST_ > 0.45 eV), in comparison with kT [6]. In contrast, it can be observed in some xanthene dyes and a few ketones and porphyrins [7,8,9]. Owing to its application to organic light-emitting diodes (OLED), there has been a recent burst of interest on TADF, with the synthesis of many new molecules having this property [10]. Fullerenes, especially C_70_, have an extraordinary TADF efficiency as shown in the singlet–triplet interconversion diagram in [11]. Owing to the long lifetime of the triplet state (millisecond range) and broad TADF temperature range (from −60 °C up to at least 100 °C) of C_70_ and derivatives, they have been applied in sensitive oxygen (in the ppmv range) and temperature optical sensors [12,13]. In 2011, Berberan-Santos and co-workers discovered the carbon-13 (C-13) isotope effect in luminescence (not observed before in any molecule), and in particular in C_70_: the triplet lifetime of C-13 C_70_ is approximately double that of normal (C-12) C_70_. This implies that TADF efficiency is much higher in C-13 C_70_, making it the brightest fullerene [14], and allowing its use in a highly sensitive optical oxygen sensor (ppbv range) [15]. TADF studies in suitable polymer matrices are limited by the maximum temperature for which matrices are still rigid (glass transition temperature) or chemically stable, e.g., ca. 100 °C for polystyrene) [16]. In order to work significantly above 100 °C while retaining rigidity and chemical stability, new matrices are needed.

Calixarenes [17] are a versatile class of macrocyclic compounds widely used in supramolecular chemistry. They are synthesized by base-catalyzed condensation reaction of a *p*-substituted phenol (usually *p-tert*-butylphenol) and formaldehyde, and they can be readily functionalized at both the upper and lower rims. Due to their high level of pre-organization and various cavity sizes, they can bind cationic, anionic, and neutral species. Parent calixarenes (those with free hydroxyl groups at the lower rim) have a bowl shape cavity with internal π-electrons, which may indicate good host properties for spherical guests with external π-electrons, such as fullerenes. These calixarenes are also characterized by high melting points, usually above 250 °C.

**Figure 1:**
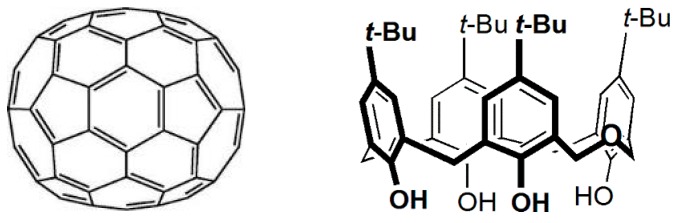
Structures of fullerene C_70_ and of *p*-*tert*-butyldihomooxacalix[4]arene.

The interactions of several calix[*n*]arenes (*n* = 4, 5, 6, 8) with C_60_ and C_70_, either in the solid state or in solution, have been extensively investigated [18,19]. The stoichiometry of the solid state complexes is mainly 1:1 (host:guest), although a few 1:2 and 1:3 complexes have been reported with some calix[6]arenes. In solution, the binding constants of the 1:1 complexes in various solvents have been determined by different methods, such as UV–vis absorbance, fluorescence, and ^1^H NMR titrations. Herein, we use a dihomooxacalix[4]arene (calix[4]arene analogues in which one CH_2_ bridge is replaced by one CH_2_OCH_2_ group) [20] (Figure 1), as a solid matrix for high temperature measurements and study. For the first time, the TADF of normal and C-13 enriched fullerene C_70_ up to 140 °C using *p-tert*-butyldihomooxacalix[4]arene (DHOC4) [21,22] with a melting point of 350 °C should allow attaining even higher temperatures.

## 2. Results and Discussion

**Figure 2:**
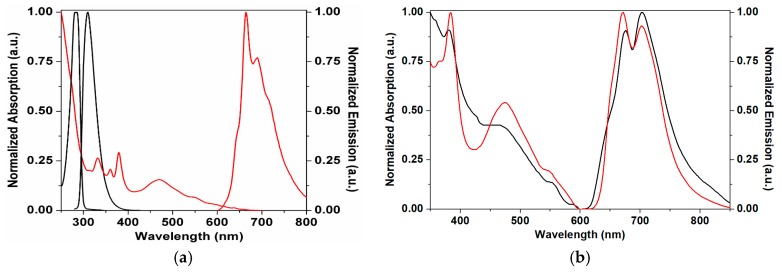
Absorption and emission spectra of DHOC4 (black line) and ^12^C_70_ (red line) in dichloromethane (**a**) and of ^12^C_70_ in DHOC4 (black line) and PS (red line) (**b**).

The absorption and emission spectra of free DHOC4, ^12^C_70_, ^12^C_70_ in DHOC4 and polystyrene (PS) matrices [16] are shown in Figure 2. The scattering by the DHOC4 matrix is apparent, otherwise the same vibronic bands are observed in both cases. The DHOC4 itself absorbs only below 320 nm. The emission spectrum of ^12^C_70_ in DHOC4 is also similar to that in PS. This is consistent with a homogeneous dispersion of C_70_ in the DHOC4 matrix and a weak electronic interaction. The C-13 enriched fullerene has absorption and emission spectra identical to those of ^12^C_70_, as expected [14,16].

In Table 1, the fluorescence and delayed fluorescence lifetimes of ^12^C_70_ and ^13^C_70_ in DHOC4 and PS are presented.

The fluorescence lifetimes (Table 1) are similar in both matrices. On the other hand, delayed fluorescence lifetimes in calixarene are about 20% lower. This difference probably reflects a stronger interaction of the fullerene with the slightly polar calixarene matrix that nevertheless does not quench either fluorescence nor phosphorescence.

**Figure 3:**
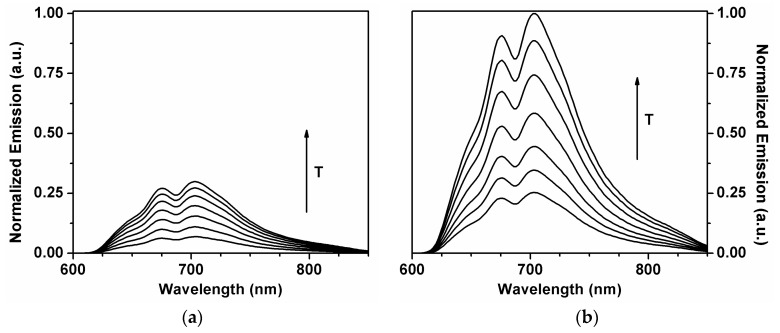
Dependence of TADF emission with the temperature for ^12^C_70_ (**a**) and ^13^C_70_ (**b**) in DHOC4 host medium.

TADF of C_70_ in the calixarene solid matrix was measured between 20 °C and 140 °C. In Figure 3, the TADF dependence with the temperature is presented, for both ^12^C_70_ and ^13^C_70_. All measurements were made with a degassed sample, except for one measurement at 25 °C used as an intensity reference. A strong temperature dependence is observed for both ^12^C_70_ and ^13^C_70_ meaning that fullerene TADF is much stronger than fullerene prompt florescence. After degassing the ^12^C_70_-DHOC4 sample, an increase of 7.4-fold in the fluorescence intensity was observed at 25 °C, while for ^13^C_70_-DHOC4 the increase in fluorescence intensity was 31-fold.

This difference between ^12^C_70_ and ^13^C_70_ reflects the isotope effect, as discussed in [12,14]. The fluorescence intensity ratios I_DF_/I_PF_ are consistently lower than those measured in PS films [16]. This difference may result from the stronger fullerene-matrix interaction, as mentioned, a lowering of the TADF lifetime being the main effect. DHOC4-fullerene interaction, of the host–guest type, differs from that in polymer matrices (PS, Zeonex, Paraffin, P1VN, PtBMA) previously used [2,3,16].

Table 2 summarizes the measured I_DF_/I_PF_ values for ^12^C_70_ and ^13^C_70_ in DHOC4 and compares them with ^12^C_70_ and ^13^C_70_ in PS [16].

The maximum temperature attained for most of the TADF studies with PS is about 100 °C, owing to the glass transition temperature (T_g_ = 107 °C) [23]. Notwithstanding, in a recent study of TADF in porphyrins using PS as a matrix, measurements up to 130 °C were reported [24]. Nevertheless, measurements well above the glass transition temperature imply structural changes in the film, including rigidity loss. Most of the polymers used (PS, Zeonex, P1VN, PtBMA) have a T_g_ below 120 °C. On the other hand, using DHOC4 as the matrix allows attaining much higher temperatures. With our experimental setup (heating power vs. thermal insulation) a maximum of 140 °C was reached, but much higher values are, in principle, possible.

**Figure 4:**
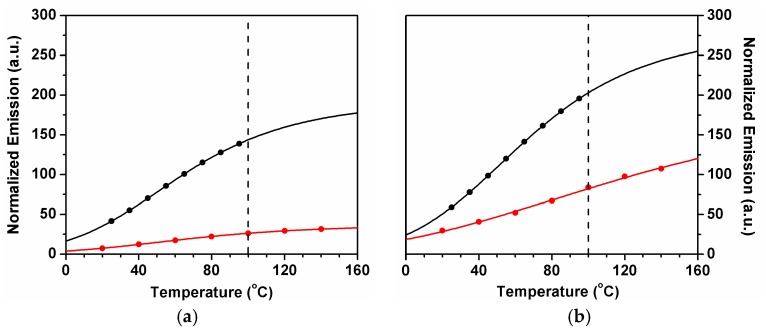
I_DF_/I_PF_ versus temperature for ^12^C_70_ (**a**) and ^13^C_70_ (**b**) in DHOC4 (red) and PS (black). The lines correspond to Equation (2).

In order to determine the TADF parameters an approach already applied to the studies of C_70_ in paraffin [2] and in PS [16,25] was used. This method is based on the measurement of the prompt fluorescence (PF) and delayed fluorescence (TADF) steady-state intensities, I_PF_ and I_DF_, as a function of temperature, plotted according to

(1) $$\ln\left[\frac{I_{\mathrm{PF}}}{I_{\mathrm{DF}}}-\left(\frac{1}{\phi_{T}}-1\right)]=\ln[\frac{1}{\phi_{T}}\left(\frac{1}{\phi_{S}^{\infty }}-1\right)\right]+\frac{\Delta E_{\mathrm{ST}}}{\mathrm{RT}}$$

where $\phi_{T}$ is the triplet formation quantum yield, $\phi_{S}^{\infty }$ is the singlet formation quantum yield [2] extrapolated to high temperatures, and ΔE_ST_ is the effective singlet–triplet energy gap. From Equation (1), it is therefore possible to obtain ΔE_ST_ from the temperature dependence of the I_DF_/I_PF_ ratio. The shape of the plot is a sensitive function of $\phi_{T}$, not being, in general, a straight line. The best value of $\phi_{T}$ (assumed temperature independent) is obtained from the most linear plot. In addition to the method of Equation (1), a nonlinear fitting procedure is also possible. In Figure 4 the I_DF_/I_PF_ experimental values for ^12^C_70_ and ^13^C_70_ in DHOC4 and in PS are shown as a function of temperature. These values are also compared with the fitted values [25] obtained from:

(2) $$\frac{I_{\mathrm{DF}}}{I_{\mathrm{PF}}}={\left(a+be^{\frac{c}{T}}\right)}^{-1}$$

where

(3) $$a=\frac{1}{\phi_{T}}-1,\;b=\frac{1}{\phi_{T}}\left(\frac{1}{\phi_{S}^{\infty }}-1\right),\;c=\frac{\Delta E_{\mathrm{ST}}}{R}$$

As mentioned, from Equations (1) or (2), it is possible not only to obtain ΔE_ST_ but also $\phi_{T}$.

**Figure 5:**
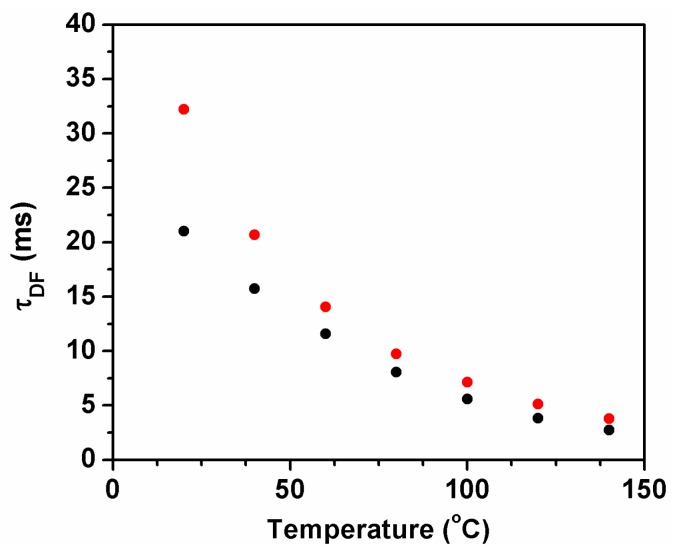
*τ*_DF_ versus temperature for ^12^C_70_ (black dots) and ^13^C_70_ (red dots) in DHOC4.

Delayed fluorescence lifetimes can also be used for the determination of TADF parameters. Palmeira and Berberan-Santos presented, in 2014, a method of analysis for the TADF of fullerene C_70_ that uses only the phosphorescence and delayed fluorescence lifetimes [16]. Delayed fluorescence lifetimes of ^12^C_70_ and ^13^C_70_ in calixarene were measured between 25 °C and 140 °C and are plotted in Figure 5.

A combination of steady-state and time-resolved data allows obtaining $\phi_{T}$ and a hypothetical phosphorescence lifetime in the absence of TADF, $\tau_{P}^{0}$ [3], using Equation (4)

(4) $$\tau_{DF}=\tau_{P}^{0}-\left(\frac{1}{\Phi_{T}}-1\right)\tau_{P}^{0}\frac{I_{\mathrm{DF}}}{I_{\mathrm{PF}}}$$

Another combination can be used to directly obtain the temperature-dependent reverse intersystem crossing rate constant from S_1_ to T_1_, $k_{ISC}^{T}$ [14,26]

(5) $$k_{ISC}^{T}=\frac{\frac{I_{\mathrm{DF}}}{I_{\mathrm{PF}}}}{\phi_{T^{\tau_{DF}}}}$$

**Figure 6:**
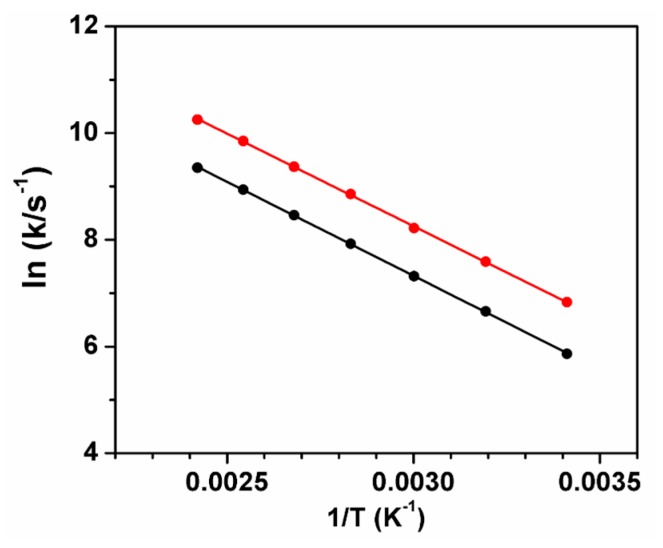
Arrhenius plot for ^12^C_70_ (black dots) and ^13^C_70_ (red dots) in DHOC4.

In this case, ΔE_ST_ is obtained from the slope of the Arrhenius plot [14], as shown in Figure 6.

The TADF parameters for ^12^C_70_ and ^13^C_70_ in DHOC4 obtained from steady-state data alone and in combination with time-resolved data are given in Table 3, along with those obtained in PS [16].

From the best fit with Equation (1), ΔE_ST_ of 26 kJ/mol for ^12^C_70_ and 23 kJ/mol for ^13^C_70_ in DHOC4 are obtained. On the other hand, using the Arrhenius plot, a common value of 29 kJ/mol is retrieved, close to that measured for PS. Using $\phi_{T}$ and τ_F_ values (Table 1), the reverse ISC rate constants ($k_{\mathrm{ISC}}^{T}$) can also be calculated (Table 3). The values are essentially independent of the C-13 enrichment degree and of the matrix used. On the other hand, the pre-exponential factor, A, that is obtained from the Arrhenius equation shows an increase of seven times and four times when going from ^12^C to ^13^C, in DHOC4 and PS, respectively.

It is important to mention that the upper limit of 140 °C reached in this study is derived from experimental constraints and not from any limitations of the photophysics of the host or guest matrix stability.

## 3. Materials and Methods

^12^C_70_ 85+% and ^13^C_70_ (85% carbon-13 enriched) 95+% were purchased from MER Corporation (Arizona, AZ, USA). Toluene (spectroscopic grade) and chloroform (spectroscopic grade) were purchased from Sigma-Aldrich (St. Louis, MO, USA). *p-tert*-butyldihomooxacalix[4]arene was synthesized according to [27]. The fullerene-DHOC4 solid was prepared by dissolving 20 mg of the corresponding DHOC4 in toluene (0.9 g) at 50 °C. After being completely dissolved, the solution was allowed to cool down and then 0.35 mL of a 7.5 × 10^−4^ M fullerene solution in toluene was added. The mixture was stirred during a few minutes and then dried using nitrogen gas. After complete drying 1 mL of chloroform was added, redissolving the solid. This solution was finally spread onto a quartz plate at room temperature. After complete evaporation, the plate containing the fullerene dispersed in solid calixarene was placed in a quartz cell that was degassed (final pressure: 1.5 × 10^−7^ atm), the cell being sealed afterwards. Absorption spectra were recorded on a UV-3101PC UV–vis–NIR spectrophotometer (Shimadzu, www.shimadzu.com, Kyoto, Japan). TADF spectrum was obtained with a Fluorolog F112A fluorimeter (Spex, www.jobinyvon.com), in the front face configuration, with an excitation wavelength of 470 nm and 4.5 nm excitation and emission slits. A band pass filter was used in the excitation and a cut-off filter (600 nm) in the emission. Emission spectra were not corrected for the spectral response of the optics and photomultiplier. Time-resolved picosecond fluorescence intensity decays were obtained by the single-photon timing method with laser excitation, with the set-up described in [28].

## 4. Conclusions

A study of the fullerene C_70_ TADF dependence using DHOC4 as a high temperature matrix was presented for the first time, being observed up to 140 °C. Owing to absence of T_g_ and high melting points, calixarenes are thus introduced as suitable alternative matrices for high temperature TADF studies and applications.

## Figures and Tables

**Table 1:** Fluorescence (*τ*_F_) and delayed fluorescence (*τ*_DF_) lifetimes of ^12^C_70_ and ^13^C_70_ in DHOC4 and PS at room temperature.

	^12^C_70_		^13^C_70_	
Matrix	DHOC4	PS	DHOC4	PS
τ_F_ (ns)	0.54	0.63	0.62	0.63
τ_DF_ (ms)	20	24	32	41

**Table 2:** I_DF_/I_PF_ values of ^12^C_70_ and ^13^C_70_ in DHOC4 and PS.

	^12^C_70_		^13^C_70_	
T (°C)/Matrix	DHOC4	PS	DHOC4	PS
25	8.54	40.5	31.4	58.9
60	17.5	93.3	54.3	131
95	25.3	139	79.2	198
140	31.4	—	109	—

**Table 3:** Photophysical parameters of ^12^C_70_ and ^13^C_70_ in DHOC4 and PS.

	^12^C_70_		^13^C_70_	
Matrix	DHOC4	PS	DHOC4	PS
ΔE_ST_ (kJ/mol) ^1^	26	29	23	28
ΔE_ST_ (kJ/mol) ^2^	29	31	29	31
ϕ_T_^1^	0.973	0.995	0.994	0.997
ϕ_T_^3^	0.971	0.995	0.992	0.996
$k_{ISC}^{T}$ (s^−1^) ^2^	1.8 × 10^9^	1.6 × 10^9^	1.6 × 10^9^	1.6 × 10^9^
A (s^−1^) ^2^	5.9 × 10^7^	4.0 × 10^8^	1.2 × 10^8^	4.8 × 10^8^
$\tau_{P}^{0}$ (ms) ^3^	25	31	33	51

^1^ from Equation (1). ^2^ from Equation (5). ^3^ from Equation (4).
